# Systematic Review of Preclinical Studies on the Efficacy and Mechanisms of Herbal Medicines in Post-Myocardial Infarction Heart Failure with Reduced Ejection Fraction

**DOI:** 10.3390/medicina60071101

**Published:** 2024-07-05

**Authors:** Soyeong Yun, Jieun Oh, Hongmin Chu, Dasol Park, Jungtae Leem

**Affiliations:** 1 Department of Obstetrics & Gynecology of Korean Medicine, Wonkwang University Jeonju Korean Medicine Hospital, 99 Garyeonsan-ro, Deokjin-gu, Jeonju 54887, Jeollabuk-do, Republic of Korea; yjw6631@naver.com; 2 Department of Neuropsychiatry of Korean Medicine, Wonkwang University Jeonju Korean Medicine Hospital, 99 Garyeonsan-ro, Deokjin-gu, Jeonju 54887, Jeollabuk-do, Republic of Korea; wldms2531@naver.com; 3 Department of Internal Medicine and Neuroscience, College of Korean Medicine, Wonkwang University, 460 Iksan-daero, Sin-dong, Iksan 54538, Jeollabuk-do, Republic of Korea; hongminchu2@gmail.com; 4 Department of Diagnostics, College of Korean Medicine, Wonkwang University, 460 Iksan-daero, Sin-dong, Iksan 54538, Jeollabuk-do, Republic of Korea; 5 Research Center of Traditional Korean Medicine, College of Korean Medicine, Wonkwang University, 460 Iksan-daero, Sin-dong, Iksan 54538, Jeollabuk-do, Republic of Korea; 6 Korean Medicine Clinical Research Institute, Wonkwang University Korean Medicine Hospital, 895 Muwang-ro, Iksan 54538, Jeollabuk-do, Republic of Korea

**Keywords:** heart failure with reduced ejection fraction, cardiovascular disease, time-dependent, immunohistological change, traditional herbal medicine, mechanism, animal experiment

## Abstract

*Background and Objectives*: Heart failure with reduced ejection fraction (HFrEF) remains a significant burden. Traditional herbal medicines have shown cardioprotective effects in treating HFrEF. However, the implications of herbal formulation considering the dynamic immunohistological changes in the myocardium following acute ischemic injury have been insufficiently discussed. This review investigated the efficacy and mechanisms reported in studies using rat or mouse models of HFrEF induced by left descending coronary artery ligation. *Materials and Methods*: A systematic search was conducted using PubMed, Embase, AMED, CINAHL, and CENTRAL databases. Information was extracted regarding study characteristics, disease model induction protocols, intervention characteristics, treatment protocols, outcomes, and suggested mechanisms. Hierarchical cluster analysis of test drugs was performed based on constituent herb similarities. The risk of bias (RoB) was assessed using the Systematic Review Center for Laboratory animal Experimentation RoB tool. *Results*: Overall, 26 studies met the eligibility criteria. HF model induction periods after LADCA ligation ranged from 1 day to 12 weeks. Most studies administered the test drug for four weeks. Commonly used herbs included *Panax ginseng*, *Astragalus membranaceus*, *Salvia miltiorrhiza*, *Carthamus tinctorius*, and *Lepidium apetalum*, which demonstrated anti-fibrotic, anti-inflammatory, and anti-apoptotic effects through various signaling pathways. The overall RoB was relatively high. No significant association was found between model induction periods and herbal formulations or examined mechanisms. *Conclusions*: Future research should consider the time-dependent immunohistological features of the myocardium during HF treatment.

## 1. Introduction

Heart failure (HF) is a clinical syndrome resulting from structural or functional disorders affecting ventricular contractility. The 2022 American Heart Association (AHA)/American College of Cardiology (ACC)/Heart Failure Society of America (HFSA) guidelines introduced a universal classification for serial assessment and reclassification based on changes in left ventricular ejection fraction (LVEF), highlighting the dynamic nature of HF [1,2]. Despite medical advancements, HF continues to impose significant medical and socioeconomic burdens. In 2017, the global patient population was estimated at 64.3 million [3]. While the incidence of HF has decreased, its prevalence is rising due to population aging. Morbidity and mortality associated with HF remain high, and the quality of life of affected patients is poor [4]. Moreover, all cardiovascular diseases such as hypertension, atherosclerosis, and atrial fibrillation can progress to heart failure if not properly managed. Thus, research on heart failure therapies remains crucial.

Ischemic cardiomyopathy, particularly following myocardial infarction (MI), is a major risk factor for heart failure with reduced ejection fraction (HFrEF) [5]. HF complications during hospitalization for acute MI are significantly associated with higher mortality and poorer prognosis [6,7]. Furthermore, lower LVEF values in chronic HF patients are linked to higher treatment costs [8]. Current guideline-directed medical therapy (GDMT) for HFrEF has demonstrated improved clinical outcomes [9]. However, challenges such as low adherence and suboptimal dosages due to patient frailty, complex comorbidities, perceived side effects, drug ineffectiveness, and cost, as well as healthcare professional and institutional factors, indicate the need for alternative and complementary treatment options [10]. Recently, results from a large-scale randomized clinical trial on Qiliqiangxin for HFrEF were presented at the European Society of Cardiology congress. This trial, based on a decade of prior clinical and preclinical studies, garnered attention from the United States and Europe [11,12]. Indeed, herbal medicine treatments for heart failure are gaining global recognition beyond East Asia. Various herbal medicines targeting different mechanisms are being researched, developed, and applied in clinical settings. 

In treating HF, considering dynamic changes in the myocardium over time and the association between LVEF values and prognosis is crucial [9]. Accordingly, studies exploring the efficacy and mechanisms of EATM interventions, considering the immunohistological changes in the myocardium over time, are important. Herbal formulations have been prescribed widely in East Asia for HF treatment based on syndrome differentiation, focusing on “yang deficiency”, “yin deficiency”, “qi deficiency”, “water retention”, “turbid phlegm”, and “blood stasis” [13]. Additionally, studies have examined the effects of herbal medicines on HF using real-world data from Taiwan, China, and Japan [14,15]. Reviews have been conducted on the efficacy and mechanisms of EATM in HF, heart failure with preserved ejection fraction (HFpEF), and the anti-inflammatory effects of EATM in post-MI HFrEF [16,17,18]. However, research into the optimal combination of herbal medicines and their mechanisms according to the disease phase in treating HF, especially following MI, remains limited. 

This review highlights the importance of considering a time-dependent approach in herbal medicine treatment for HF, focusing on the immunohistological changes in myocardial tissue over time in cardiovascular diseases. It also aims to identify unexplored research areas to aid in planning and designing future research projects. Therefore, we systematically reviewed the experimental studies on oral herbal medicines in HF murine models induced by left anterior descending coronary artery (LADCA) ligation. Inclusion was limited to the research in which animal subjects were confirmed with an LVEF of <50% to ensure successful HFrEF model induction. We summarized and reviewed the formulation of the herbal medicines used, the period of model induction, the timing of treatment initiation, and treatment duration, as well as the effects and mechanisms explored in the research.

## 2. Materials and Methods

### 2.1. Study Registration

The protocol for this systematic review was registered with the OSF (https://osf.io/2jgc4/ (accessed on 3 April 2024)), and the study followed the Preferred Reporting Items for Systematic Reviews and Meta-Analyses (PRISMA) guidelines. 

### 2.2. Data Sources and Search Strategy

A systematic search was conducted using PubMed, Embase, AMED, CINAHL, and CENTRAL databases. Two researchers (SY and JO) conducted the initial search from 30 May 2022 to 1 June 2022. The second search was conducted from 22 December 2023 to 1 January 2024. There were no restrictions on language, publication date, or publication status. The search terms “heart failure” and “herbal medicine” were used, and the exact search strategy for each database is presented in Table S1.

### 2.3. Eligibility Criteria

Studies that induced MI in rats or mice by permanently ligating the LADCA and had subjects with an LVEF of less than 50% were selected. Selected studies were limited to the ones using herbal medicine as the test drug, administered orally or via the intragastric route to subjects. 

#### 2.3.1. Animals

Inclusion criterion:

The HFrEF animal model using rats or mice, in which MI was induced through the permanent ligation of the LADCA, subsequently confirmed to have an average LVEF of <50%.

Exclusion criteria:

- Studies not utilizing the murine model.
- Disease models induced by methods other than permanent LADCA ligation.
- Studies without records of criteria for the success of model induction with an average LVEF of <50%.

#### 2.3.2. Interventions

Inclusion criterion: enteral administration of herbal medicine (e.g., by gavage, oral administration, or intragastric irrigation).

Exclusion criterion: non-herbal medicine or non-enteral administration (e.g., intraperitoneal injection).

#### 2.3.3. Comparisons

No restrictions.

#### 2.3.4. Outcome Measures

No restrictions.

#### 2.3.5. Study Designs

Inclusion criterion: in vivo studies testing efficacy.

Exclusion criteria:

- Toxicological, pharmacodynamic, pharmacokinetic, in vitro, and in silico studies.
- Human clinical studies.
- Reviews.

#### 2.3.6. Other Limitations

Inclusion criterion: original articles.

Exclusion criterion: conference abstracts, surveys, editorials, consensuses, and letters.

### 2.4. Data Extraction

Two researchers (SY and JO) independently performed data extraction. The extracted information included the following:

- Baseline study characteristics: author and year of publication.
- Animal characteristics: species and strain, sex, weight, and age.
- Protocol of the post-MI HFrEF disease model induction: LADCA ligation method, model induction period, and the LVEF measurements of the animals after model induction or the LVEF values used as criteria for successful model induction.
- Intervention characteristics and treatment protocol: name and herbal formulation of the test drug, positive control drug, drug preparation method, dose of drugs, administration route, treatment frequency, treatment duration, and the number of allocated animals for each study group.
- Outcomes and suggested mechanisms of action.

In cases where both in vivo and in vitro experiments were conducted, the in vitro experimental data were not extracted. Any disagreements were resolved by seeking the opinions of a third researcher (JL). The names of the herbs of the test drugs were denoted by scientific names based on the Medicinal Plant Names Services database (https://mpns.science.kew.org) as of 24 April 2024 [19]. 

### 2.5. Hierarchical Cluster Analysis

Herbal medicines from the included studies were presented in a dendrogram after hierarchical cluster analysis based on the similarity of constituent herbs. Hierarchical cluster analysis was conducted using the *ggtree* package in RStudio (v.2023.06.0; Posit Team. RStudio: Integrated Development Environment for R; Posit Software, v.2023.06.0 PBC; Boston, MA, USA) [20].

### 2.6. Quality Assessment

The risk of bias (RoB) was assessed using the Systematic Review Center for Laboratory Animal Experimentation (SYRCLE) Risk of Bias tool, which comprises ten items for animal intervention studies. Two researchers (SY and JO) independently assessed the methodological quality of the selected studies. Any disagreements were resolved by seeking the opinions of a third researcher (JL). 

## 3. Results

### 3.1. Search Results

**Figure 1:**
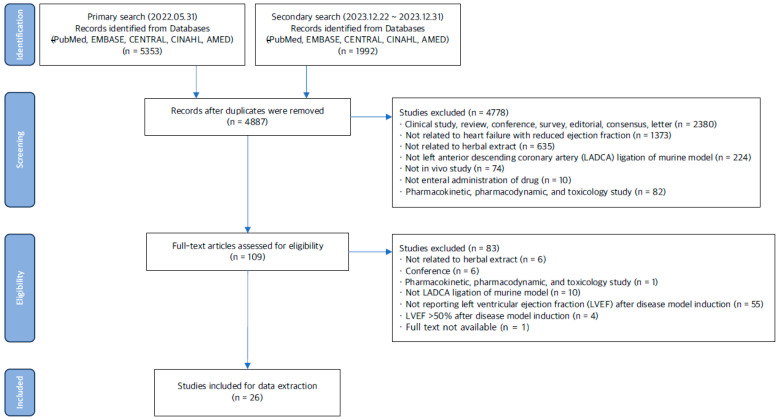
Flow diagram of study search.

The search results are presented in Figure 1. A total of 5353 potentially relevant articles were identified from the initial search conducted from 31 May to 1 June 2022. A subsequent search from 23 December 2023 to 1 January 2024 found an additional 1992 articles published between 1 May 2022 and 31 December 2023. After removing duplicates, 4887 articles remained. Furthermore, 4778 articles were excluded after screening titles and abstracts. The full texts of the remaining 109 articles were accessed for eligibility, and 26 articles were ultimately selected for review. 

### 3.2. Characteristics of the Included Studies

The characteristics of the included studies are presented in Table 1. 

#### 3.2.1. Animal Species

Among the 26 studies, 21 used Sprague Dawley male rats, 3 used Wistar male rats, 1 used C57BL/6 male mice, and 1 used C57 male mice. The weight ranges of rodents used in the studies were 160–280 g for Sprague Dawley rats, 200–300 g for Wistar rats, 20–25 g for C57BL/6 mice, and 20–23 g for C57 mice. Most studies did not record the exact age of the mice or rats. 

#### 3.2.2. HF Model Induction Period after LADCA Ligation

In all studies, acute MI was induced via LADCA ligation, followed by a period after which LVEF was measured to confirm the successful induction of the HFrEF model. The induction period varied, with ten studies using a four-week period, seven studies using a one-week period, and four studies using a two-week period. The shortest induction period recorded was one day, and the longest was 12 weeks. One study varied the start date of drug administration among the experimental groups to be one day, two weeks, four weeks, and six weeks after ligation [21]. One study did not record the period from LADCA ligation to the start of treatment [22].

**Table 1:** Research characteristics of included studies.

Study	Test Drug	Herbal Composition of Test Drug	Animal (1) Species (2) Sex (3) Weight (g) (4) Age	Model Induction Period	LVEF after Successful Modeling	Treatment Duration	Effects and Suggested Mechanism
Zeyu Zhang, 2023 [23]	Optimized new Shengmai powder (ONSMP)	*AM1*, *CP1*, *ES1*, *SM*, *TS*, *PC*, *LA*, *OJ*, *CA1*	(1) SD rats (2) male (3) 200 ± 20 (4) *ND*	12wks	≤50%	4 wks	Antifibrosis and ↓ MAPK pathway
Zhou Zhou, 2023 [24]	*Carthamus tinctorius* L. and Lepidium apetalum Willd. herbal solution (CL)	*CT*, *LA*	(1) SD rats (2) male (3) 190–210 (4) *ND*	1 wk	<50%	4 wks	Antifibrosis and ↓ TGF β1/Snail Pathway
Ye Li, 2023 [22]	Shengmai Yin (SMY)	*PG1rg*, *OJ*, *SC*	(1) SD rats (2) male (3) 160–180 (4) *ND*	*ND*	Negative control: 76.78 ± 3.81% Model: 35.45 ± 12.46% Positive control: 47.41 ± 9.48% SMY-L: 39.14 ± 7.78% SMY-H: 47.54 ± 8.89%	4 wks	Antiautophagy and ↑ Akt/mTOR pathway
Yan-Ni Su, 2023 [25]	Xinfuli granule (XG)	*AM1*, *PG1*, *SM*, *PC*, *OJ*	(1) SD rats (2) male (3) 210 ± 10 (4) *ND*	4 wks	<40% Model: 38.4 ± 5.1% Sham: 77.5 ± 2.5%	4 wks	Anti-apoptosis and ↓ RHOA/ROCK pathway
Xiaoyu Tian, 2023 [26]	Xinbao Pill (XBP)	*DM*, *PN*, *PG1*, *AC*, *CC1*, *CN*, *BBG*, *BS*, *MM*	(1) SD rats (2) male (3) 200 ± 20 (4) *ND*	4 wks	<45%	4 wks	Neurohumoral modulation and ↓ USP18 and MDM2/β-arrestin2/Nedd4
Xiao-Hong Wei, 2023 [27]	XinLi formula (XLF)	*PA*, *AM1*, *PG1*, *CP2*, *CO*	(1) SD rats (2) male (3) 180 ± 10 (4) 8 wks	1 wk	<45%	4 wks	Anti-inflammation and ↓ AGTR1/NLRP3 pathway ↓ AGTR1-AQP1 interaction
Shuai Wang, 2023 [28]	optimized new Shengmai powder (YHXSMS)	*AM1*, *CP1*, *ES1*, *TS*, *DS*, *PC*, *OJ*, *CA1*, *SM*	(1) Wistar rats (2) male (3) 200–240 (4) *ND*	6 wks	≤45%	4 wks	Anti-apoptosis and ↓ UPS pathway
Jianhua Li, 2023 [29]	Yixin Granule (YX)	*AM1*, *AA*, *BC*, *PG2*, *PP*, *SM*, *AM2*, *CC1*	(1) SD rats (2) male (3) 220–240 (4) *ND*	5 wks	≤45% on the last day of 4 wks after surgery	4 wks	Antifibrosis and ↓ ADAMTS8 expression
Fengrong Zhang, 2023 [21]	Xin-shu-bao tablet (XSB)	*SM*, *PL*, *ES1*, *CA2*, *CP3*	(1) C57 mice (2) male (3) 20–23 (4) adult	1 day 2 wks 4 wks 6 wks	<40% at 4 wks after MI	8 wks 6 wks 4 wks 2 wks	Antifibrosis and Not specified
Xiaofei Chen, 2022 [30]	Renshen-Fuzi herb pair (RS-FZ)	*PG1*, *AC*	(1) SD rats (2) male (3) 220 ± 10 (4) *ND*	4 wks	<50%	4 wks	Antifibrosis and Not specified
Jin Ma, 2022 [31]	Danqi soft capsule (DQ)	*PN*, *SM*	(1) SD rats (2) male (3) 250–280 (4) *ND*	1 wk	<45%	4 wks	Antifibrosis and ↓ TGF β1/p-Smad 3 pathway ↑ Cx43
Yanyan Wang, 2020 [32]	Qiliqiangxin (QL)	*AM1*, *AC*, *PG1*, *SM*, *LA*, *PS*, *APA*, *CT*, *PO*, *CR*, *CC1*	(1) SD rats (2) male (3) 200 ± 20 (4) adult	2 wks	<50%	6 wks	Angiogenesis Energy metabolism (glucose) and ↑ HIF-1α/VEGF pathway HIF-1α-independent pathway
CHEN Jiaxian, 2020 [33]	Qisheng-Yiqi Dropping Pill (QSYQ)	*AM1*, *SM*, *PN*	(1) SD rats (2) male (3) 220 ± 20 (4) 6–7 wks	1 day	≤50%	4 wks	Anti-apoptosis and ↑ Nrf2/HO-1 pathway
Binhao Shi, 2020 [34]	Qi Dan Li Xin pill (QD)	*SM*, *AM1*, *LA*, *PC*, etc.	(1) SD rats (2) male (3) 220–240 (4) *ND*	4 wks	38–50%	4 wks	Anti-apoptosis Proautophagy and ↓ mTOR/p70S6k signaling pathway
Qifei Zhao, 2019 [35]	Qiliqiangxin (QLQX)	*AM1*, *AC*, *PG1*, *SM*, *LA*, *PS*, *APA*, *CT*, *PO*, *CR*, *CC1*	(1) SD rats (2) male (3) 200–220 (4) *ND*	4 wks	<50%	4 wks	Anti-apoptosis and ↑ PI3K/AKT/↓ GSK3β signaling pathway
Lijun Zhang, 2019 [36]	Ginkgo biloba extract (GBE)	*Ginkgo biloba* L.	(1) C57BL/6 mice (2) male (3) 20–25 (4) 8–10 wks	2 wks	<30%	4 wks	Anti-apoptosis Anti-inflammation and Not specified
Xu Yan, 2018 [37]	Shenfu Formula (Shenfu)	*PG1*, *AC*	(1) SD rats (2) male (3) 250 ± 20 (4) adult	1 wk	Model: 34.57 ± 6.16% Shenfu: 33.63 ± 6.30%	30 days	Anti-apoptosis and ↓ Fas, FasL ↑ Bcl-2/Bax
Junzeng Fu, 2018 [38]	Qiliqiangxin (QL)	*AM1*, *AC*, *PG1*, *SM*, *LA*, *PS*, *APA*, *CT*, *PO*, *CR*, *CC1*	(1) SD rats (2) male (3) 200–220 (4) *ND*	5 wks	≤45%	4 wks	Anti-inflammation Energy metabolism (Lipid) Promote endothelial function and Not specified
Jin Ma, 2018 a [39]	Shengsong Yangxin capsule (SSYX)	*PG1*, *OJ*, *NJ*, *TE*, *ZJ*, *CO*, *SM*, *PL*, *SS*, *GY*, *ES2*, *CC2*	(1) SD rats (2) male (3) 250–280 (4) *ND*	1 wk	<45%	4 wks	Antifibrosis Anti-fibrillation and ↓ TGF-β1 pathway
Jin Ma, 2018 b [40]	Fumai granule (FM)	*PG1*, *OJ*, *SC*, *AS*, *BG*, *GU*	(1) SD rats (2) male (3) *ND* (4) *ND*	1 wk	<45%	4 wks	Antifibrosis Anti-fibrillation and ↓ TGF-β1 pathway
Guozhen Yuan, 2018 [41]	Bao Yuan Tao Hong (BYTH)	*PG1*, *AM1*, *CC1*, *GU*, *RG*, *AS*, *PL*, *CA3*, *PP*, *CT*	(1) SD rats (2) male (3) 220–250 (4) *ND*	4 wks	Sham: 84.490 ± 7.3354% Model: 44.708 ± 8.4369% BYTH: 46.863 ± 9.0312% Valsartan: 47.911 ± 9.1068	4 wks	Antifibrosis Anti-inflammation and ↓ TGF-β1/Smad3 pathway ↓ TLR4-NF-κB pathway
Anbang Han, 2018 [42]	Qiliqiangxin (QL)	*AM1*, *AC*, *PG1*, *SM*, *LA*, *PS*, *APA*, *CT*, *PO*, *CR*, *CC1*	(1) SD rats (2) male (3) 220–250 (4) *ND*	4 wks	Sham: 84.5 ± 7.3% Model: 44.7 ± 8.4% QLQX: 47.9 ± 9.1% Valsartan: 47.9 ± 9.1%	4 wks	Antifibrosis Anti-inflammation and ↓ TGF-β1/Smad3 pathway ↓ NF-κB signaling pathway
Yunfei Qu, 2017 [43]	Shenfuqiangxin	*AK*, *PG*, *AM1*, *PO*, *OJ*, *CA3*, *SM*, *LA*, *GU*	(1) SD rats (2) male (3) 230 ± 27 (4) 45 days	2 wks	sham: 78.15 ± 5.77% model: 50.69 ± 7.13% Shenfuqiangxin: 50.24 ± 6.58	8 wks	Antifibrosis and ↓ TGF-β/Smads signaling pathway
Yaoyao He, 2015 [44]	Qili Qiangxin (QL)	*AM1*, *AC*, *PG1*, *SM*, *LA*, *PS*, *APA*, *CT*, *PO*, *CR*, *CC1*	(1) SD rats (2) male (3) 220–250 (4) *ND*	4 wks	sham: 84.490 ± 7.3354% model: 44.708 ± 8.4369% QL: 47.933 ± 9.1211% Valsartan: 47.911 ± 9.1068	4 wks	Antifibrosis Anti-inflammation and ↓ TGF-β1/Smad3 pathway ↓ TLR4 pathway
Xiao-chun Yang, 2012 [45]	Sanshen Weixin Capsula (Sanshen)	*PG1*, *SM*, *SN*	(1) Wistar rats (2) male (3) 250–300 (4) *ND*	1 wk	<45% at 3 days after surgery	5 wks	Not specified and Not specified
Yan-fang Li, 2012 [46]	Shexiangbaoxin pills (SXBXP)	*PG1*, *CC1*, *MM*, *BBG*, *BS*, *ST*, *BTD*	(1) Wistar rats (2) male (3) 250–280 (4) 10 wks	4 wks	≤45%	8 wks	Anti-apoptosis and Adrenergic receptor modulation

Abbreviations: AA, *Anemarrhena asphodeloides* Bunge; AC, *Aconitum carmichaelii* Debeaux; AK, *Aconitum kusnezoffii*; AM1, *Astragalus membranaceus* (Fisch.) Bunge; AM2, *Atractylodes macrocephala* Koidz.; *APA, Alisma plantago-aquatica* subsp. *orientale* (Sam.) Sam.; AS, *Angelica sinensis* (Oliv.) Diels; BBG, *Bufo bufo gargarizans* Cantor (venom glands); BC, *Bupleurum chinense* DC.; BG, *Bitter ginseng* (not specified); BS, *Borneolum syntheticum*; BTD, *Bos taurus domesticus* (Gallstone); CA1, *Citrus* × *aurantium* L.; CA2, *Curcuma aromatica* Salisb.; CA3, *Conioselinum anthriscoides* “Chuanxiong”; CC1, *Cinnamomum cassia* Presl; CC2, *Coptis chinensis* Franch.; CN, *Cervus nippon* Pantotrichum (young horn); CO, *Cornus officinalis* Siebold & Zucc.; CP1, *Codonopsis pilosula* (Franch.) Nannf; CP2, *Curcuma phaeocaulis* Valeton; CP3, *Crataegus pinnatifida* Bunge; CR, *Citrus reticulata* Blanco; CT, *Carthamus tinctorius* L.; DM, *Datura metel* L.; DS, *Descurainia sophia* (L.) Webb ex Prantl; ES1, *Eleutherococcus senticosus* (Rupr. & Maxim.) Maxim.; ES2, *Eupolyphaga sinensis* (Walker); GY, *Goniophlebium yunnanense* (Franch.) Bedd.; GU, *Glycyrrhiza uralensis* Fisch. ex DC.; HIF-1α, hypoxia-inducible factor 1-alpha; LA, *Lepidium apetalum* Willd.; LVEF, left ventricular ejection fraction; MAPK, mitogen-activated protein kinase; MDM2, mouse double minute 2 homolog; MI, myocardial infarction; MM, *Moschus moschiferus* L. (musk sac); ND, not described; NJ, *Nardostachys jatamansi* (D.Don) DC.; OJ, *Ophiopogon japonicus* (Thunb.) Ker Gawl.; PA, *Plantago asiatica* L.; PC, *Poria cocos* (Schw.) Wolf; PG1, *Panax ginseng* C.A.Mey; PG1rg, *Panax ginseng* C.A.Mey (Red ginseng); PG2, *Platycodon grandiflorum* (Jacq.) A.DC.; PI3K, phosphoinositide 3-kinase; PL, Paeonia lactiflora Pall.; PN, Panax notoginseng (Burkill) F.H.Chen; PO, *Polygonatum odoratum* (Mill.) Druce; PP, *Prunus persica* (L.) Batsch; PS, *Periploca sepium* Bunge; RG, *Rehmannia glutinosa* (Gaertn.) DC.; SC, *Schisandra chinensis* (Turcz.) Baill.; SD, Sprague Dawley; SM, *Salvia miltiorrhiza* Bunge; SN, *Scrophularia ningpoensis* Hemsl.; SS, *Schisandra sphenanthera* Rehder & E.H.Wilson; ST, *Styrax tonkinensis* (Pierre) Craib ex Hartwich (Resin); TGF-β1, Transforming growth factor beta 1; TE, *Taxillus estipitatus* (DC.) Danser; TS, *Trionyx sinensis* Wiegmann; UPS, ubiquitin–proteasome system; wk, week; and ZJ, *Ziziphus jujuba* Mill.

#### 3.2.3. LVEF Criterion for Successful Modeling

In ten studies, an LVEF of <45% after the induction period was established as the criterion for successful HFrEF model induction. The model induction periods for these cases ranged from one to six weeks. Six other studies set the criterion at LVEF < 50%, and in five of these studies, it was confirmed that the LVEF values before treatment initiation were, on average, within 50% after model induction. One study performed LADCA ligation in C57BL/6 mice and used an LVEF < 30% two weeks after ligation as the criterion for successful model induction [36].

#### 3.2.4. Treatment Duration

In most studies, the test drug was administered for four weeks. In two studies, the treatment duration varied among the treatment groups, ranging from two to eight weeks [21,38]. The treatment initiation date after model induction and the treatment duration were summarized and presented in Table S2. 

#### 3.2.5. Name of the Tested Herbal Medicine and Their Compositions 

**Figure 2:**
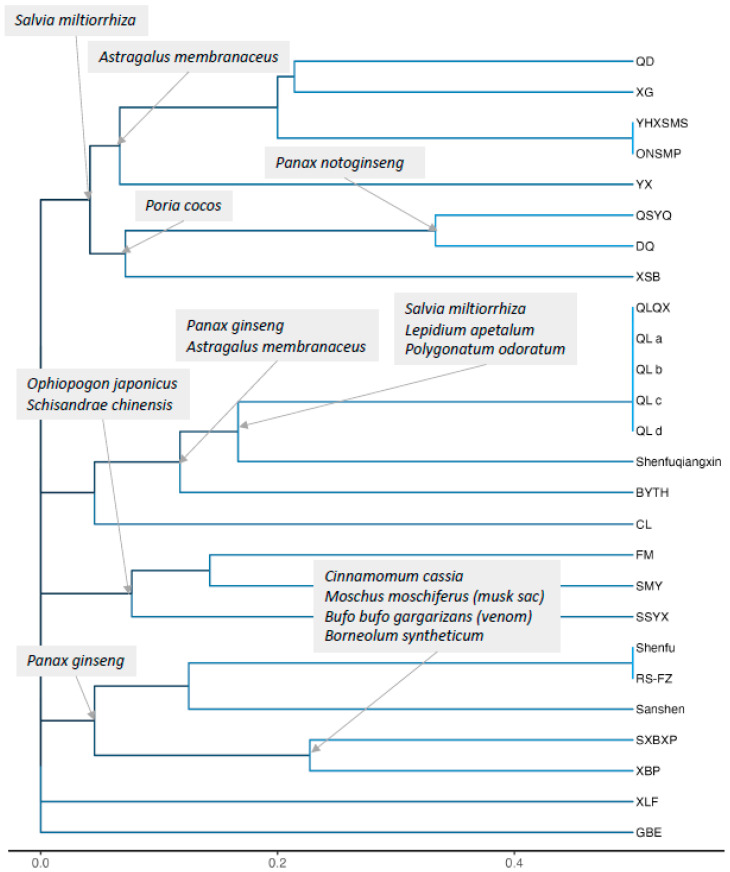
Dendrogram of herbal medicines from included studies based on the similarity of the constituent herbs. The closer two herbal medicines are to each other, the more similar the corresponding objects are. The scale at the bottom of a dendrogram is a quantitative measure that represents the level of similarity among the objects being clustered. Common herbs used within each cluster are indicated at the points where branches split. Abbreviations: BYTH, Bao Yuan Tao Hong; CL, *Carthamus tinctorius* L. and *Lepidium apetalum* Willd. herbal solution; DQ, Danqi soft capsule; FM, Fumai granule; GBE, *Ginkgo biloba* extract; ONSMP, optimized Shengmai powder; QD, Qi Dan Li Xin pill; QLQX, Qiliqiangxin; QSYQ, Qisheng-Yiqi Dropping Pill; RS-FZ, Renshen-Fuzi herb pair; Shenfu, Shenfu Formula; Sanshen, Sanshen Weixin Capsula; SMY, Shengmai Yin; SSYX, Shengsong Yangxin capsule; SXBXP, Shexiangbaoxin pills; XBP, Xinbao Pill; XG, Xinfuli granule; XLF, XinLi formula; XSB, Xin-shu-bao tablet; YHXSMS, optimized new Shengmai powder; and YX, Yixin Granule.

The herbal medicines tested in the included studies and their compositions are presented in Table S3. The source species of the herbs involved in this review are listed in Table S4. Among the included 26 studies, 20 formulations were utilized as test drugs. Qiliqiangxin was the most frequently tested and used in five studies [32,35,38,42,44], followed by optimized new Shengmai powder in two studies [23,28], and Renshen-Fuzi herb pair in two studies [30,37]. A total of 51 distinct herbs were identified in the formulations used across the included studies. *Salvia miltiorrhiza* (Dan Shen) was the most frequently used herb, appearing in 16 studies, followed by *Panax ginseng* (Ren Shen) and *Astragalus membranaceus* (Huang Qi) in 15 studies and *Schisandra chinensis* (We Wei Zi) in 11 studies. Among the 20 formulations, *Salvia miltiorrhiza* was the most commonly used, followed by *Panax ginseng*, *Astragalus membranaceus*, *Ophiopogon japonicus* (Mai Dong), *and Schisandra chinensis*. The result of the hierarchical cluster analysis based on the similarity of the constituent herbs between each formulation was displayed with a dendrogram in Figure 2. In Table S5, formulations were arranged according to their order in the dendrogram, and the constituent herbs were sorted by their frequency of inclusion in the prescriptions to facilitate the easy identification of similarities between compositions. Due to inconsistent notations of herbal component dosages across studies, dosage information was excluded from the analysis. 

#### 3.2.6. Investigated Mechanisms of Herbal Medicines in Post-MI HFrEF Treatment

The results of the in vivo experiments of the included studies were summarized in Table S6. All studies reported improvements in cardiac function through echocardiography in post-MI HFrEF animal models after administrating herbal medicines, along with serum, plasma, and histological findings related to myocardial inflammation and fibrosis. The antifibrotic effects of herbal medicines were the most researched, followed by studies on their anti-inflammatory and anti-apoptotic effects. Additional explorations were made into anti-autophagy, pro-autophagy, neurohormonal modulation, angiogenesis, glucose and lipid metabolism, anti-fibrillation effects, and regulation of angiotensin receptor expression. 

The TGF-β1/p-Smad3 pathway was the most researched mechanism for antifibrotic effects, with *Carthamus tinctorius* L. and *Lepitium aspetalum* Willd. herbal solution [24], Danqi soft capsule [31], Shengsong Yangxin capsule [39], Fumai granule [40], Bao Yuan Tao Hong [41], and Qiliqiangxin [42,44] used as test drugs. Furthermore, the antifibrotic effect through the inhibition of ADAMTS8 expression by Yixin Granule was studied [29]. The most frequently used herbs among the herbal medicines studied for their antifibrotic mechanisms were *Panax ginseng*, *Salvia miltiorrhiza*, and *Carthamus tinctorius*. 

Mechanisms for anti-apoptotic effects included the regulation of the phosphoinositide 3-kinase (PI3K)/Akt pathway by Qiliqiangxin [35], the RHOA/ROCK pathway by Xinfuli granules [25], the ubiquitin–proteasome system pathway by optimized new Shengmai powder [23], the Nrf2/HO-1 by Qisheng-Yiqi Dropping Pill [33], and mTOR/p70S6k pathway by Qi Dan Li Xin pill [34]. All five herbal medicines studied for their anti-apoptotic mechanisms contained *Salvia miltiorrhiza* and *Astragalus membranaceus*, with *Schisandra chinensis* frequently used as well.

For anti-inflammatory effects, the regulation of the AGTR1/NLRP3 pathway by Xin Li formula [27] and the TRL4-NK-κB pathway by Bao Yuan Tao Hong [41] and Qiliqiangxin [42,44] were researched. All studied herbal medicines included *Astragalus membranaceus*, with *Panax ginseng*, *Cinnamomum cassia*, and *Carthamus tinctorius* being other commonly used herbs. 

Other studies investigated the effects of HFrEF treatment mechanisms beyond the myocardium, including its effects on the hippocampus and lungs [36,41]. Additionally, research was conducted first to identify significant markers through network pharmacology or metabolomics studies and then to verify changes in these markers due to herbal medicines through in vivo studies [21,30,38]. 

The examined mechanisms, along with model induction periods and treatment durations, were presented in Table S2. No significant differences in the examined mechanisms or systematic variations in the herbal compositions of the test drugs were observed when considering the model induction period. 

### 3.3. Risk of Bias of the Included Studies

**Figure 3:**
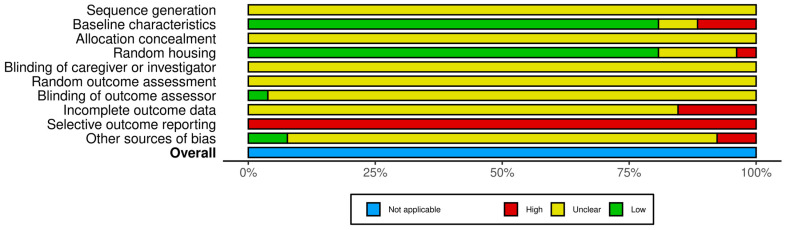
Summary plot of Systematic Review Centre for Laboratory Animal Experimentation’s risk of bias of included studies.

The summary of SYRCLE’s RoB was presented in Figure 3. None of the studies described sequence generation for random allocation or concealment. Except for one study that reported blinding of the outcome assessor, most studies did not describe the blinding of the caregivers, investigators, or assessors. While most studies did not address issues with non-random housing, they failed to describe the methods of random selection for outcome assessment or whether the outcome assessors were blinded. Published study protocols were not available for most studies. The distributions of relevant baseline characteristics such as sex, weight, and baseline LVEF of the disease model among the animal groups were balanced. Most studies did not discuss incomplete outcome data. Most studies were evaluated as free from contamination, inappropriate influence of funders, unit of analysis errors, or design-specific RoBs. 

## 4. Discussion

### 4.1. Summary of Findings

This study summarizes the cardioprotective effects of various herbal medicines in a post-MI HFrEF murine model induced by LADCA ligation through the modulation of inflammation and fibrosis-related signaling pathways. The included studies explored the effects of herbal medicines on multiple factors associated with the development of HF following myocardial injury, including myofibroblast activation, oxidative stress, proinflammatory mediators, apoptosis, and energy metabolism. However, none of the studies have considered the time-dependent immunohistological features of the myocardium during the treatment of HF with herbal medicines.

### 4.2. Comparison with Previous Studies

HFrEF is managed with GDMT, which establishes the use of four primary drug types: renin–angiotensin system inhibitors, beta-blockers, mineralocorticoid receptor antagonists, and sodium–glucose cotransporter 2 inhibitors. Due to patient, physician, or institution-related factors such as drug side effects, low adherence, and titration difficulties, GDMT is often suboptimally utilized, leading to significant health and socioeconomic impacts on affected patients [4]. Consequently, there is a need for alternative therapeutic strategies in treating HFrEF. The clinical efficacy of EATM treatments for HF has been explored in randomized controlled trials and real-world data studies, suggesting that EATM has therapeutic potential in the clinical management of HF [14,47]. 

Among the frequently used herbs in the reviewed formulations, *Salvia miltiorrhiza* and *Carthamus tinctorius* extracts via intraperitoneal injection prevented cardiac fibrosis and improved cardiac function in C57BL/6 mice in the LADCA ligation-induced MI model by inhibiting Smad3 expression [48]. Panax ginseng and its ginsenosides were also reviewed for their cardiovascular protective effects by preventing oxidative stress [49]. Additionally, administering ginseng crude extract for 14 days to Wistar rats with myocardial fibrosis induced by isopropyl adrenaline injections significantly reduced collagen fiber secretion and myocardial injury compared to the captopril group [50]. *Astragalus mongholicus* Bunge was reviewed for its anti-HF mechanisms through the regulation of the renin–angiotensin–aldosterone system, fibrosis-related signaling pathways, oxidative stress, ferroptosis, inflammation, microRNA expression, and extracellular remodeling [51]. The inhibitory effects of 2-phenylacetamide extracted from *Lepidium apetalum* Willd. on renal fibrosis via RAAS-mediated MAPK pathway and the regulation of oxidative stress have been studied in hypertension model rats [52]. Furthermore, the oligosaccharide composition of *Descurainiae sophia* used alongside *Lepidium apetalum,* as used by Ting Li Zi in EATM clinical practice, decreased serum levels of troponin I, brain natriuretic peptide, angiotensin II, aldosterone, renin, and arginine vasopressin and downregulated renal aquaporin-2 expression in Sprague Dawley rats of the doxorubicin-induced HF model, confirming its cardioprotective effect against HF [53]. The anti-HF effects summarized in this review align with the individual research findings on these herbs, presenting the therapeutic benefits of multi-target, multi-pathway strategies through the combination of these herbs. 

### 4.3. Suggestions for Future Research and Clinical Implication

After MI, a primary cause of HFrEF, the human myocardium undergoes significant immunohistological changes over several months [54]. The myocardium experiences three overlapped phases after infarction: the inflammatory, proliferative, and healing phases, with different chemokines involved in each phase [55]. Therefore, when designing studies, it is crucial to formulate hypotheses that consider the stage of the disease, as this is critical for establishing clinically optimized treatment strategies. Despite significant and continuous changes in the myocardium of rodents occurring for days and weeks following permanent LADCA ligation, the disease model induction period varied from one day to six weeks in most studies included in this review, with little discussion on the pathophysiological changes in the myocardium over time. Only one study addressed this aspect and incorporated it into its research design [21]. The multi-compound, multi-target, multi-pathway characteristics of EATM can provide a multifaceted strategy for HF but also present significant challenges in investigating optimized treatment interventions. However, research designs that consider the disease phase may overcome some of these challenges and aid in optimizing EATM intervention strategies for HF treatment and prevention. 

In post-MI HF, myocardial edema significantly affects the disease course by decreasing energy efficiency and impairing the elimination of metabolic waste products, which largely depend on vascular factors and lymphatic function [56]. The importance of research into the role of the lymphatic system in cardiovascular disease is increasingly emphasized, especially with accumulating studies on various lymph-related markers and their functions [57]. Henri et al. (2016) [58] evaluated the changes in cardiac lymphangiogenesis and related markers and cardiac water content in Wistar rats undergoing LADCA ligation. They injected VEGF-C_C152S_ into the left ventricular wall to selectively stimulate cardiac lymphangiogenesis, observing reductions in myocardial edema and fibrosis, and improvements in cardiac function. Among the frequently used herbs in the reviewed formulations, *Descurainiae sophia* demonstrated a protective effect against chronic HF through its inotropic effect and activation of the PI3K/Akt/mTOR pathway. It also exhibited a distinctive effect related to edema, such as inhibiting renal tubular reabsorption to increase urinary output and reduce pleural effusion and pulmonary edema in critically ill patients [59]. Similarly, a study on Fangjihuangqi Tang, which includes *Stephania tetrandra* (Fang Ji) with edema-reducing effects similar to those of *Descurainiae sophia* and *Lepidium apetalum*, administered to early-stage osteoarthritis mice, showed improvement in joint lymphatic drainage and alleviation of joint degeneration [60]. Herbal formulations containing herbs like *Stephania tetrandra*, *Lepidium apetalum*, or *Descuraniae sophia* could potentially demonstrate cardioprotective effects through mechanisms associated with reducing myocardial edema and enhancing the lymphatic system. If so, herbal medicines that include those capable of improving myocardial edema or cardiac lymphatic system function may provide an opportunity to further specify the optimal timing of administration for post-MI treatment. However, studies exploring the effects of herbal medicines on myocardial edema and lymphatic function in cardiovascular diseases, including MI and HF, remain scarce. Considering the dynamic stages of HF and the importance of minimizing irreversible fibrosis as an essential treatment and prevention goal, the significance of researching herbal combinations and optimizing the timing of administration based on therapeutic targets tailored to these stages is paramount, and further studies are urgently required. 

Furthermore, research designs that consider pathophysiology-targeted herbal formulas should be actively pursued, not only for ischemic heart disease but also for left ventricular dysfunction (LVD) caused by non-ischemic factors. Clinical studies have demonstrated that proprietary Chinese medicine, used as adjunctive therapy, improved symptoms, exercise capacity, and serum markers of microvascular function in patients with microvascular angina, indicating the clinical effectiveness of herbal medicines for LVD caused by non-obstructive coronary artery disease [61]. In the clinical setting of EATM, where practitioners more frequently encounter transient LVD due to non-obstructive causes, understanding the various underlying mechanisms and factors associated with myocardial recovery is critical, as is applying timely diagnosis and treatment [62]. However, research designs that specifically consider the diverse pathological mechanisms and the time variable in herbal medicine studies are severely lacking, a gap that urgently needs to be addressed. 

### 4.4. Strengths and Limitations

Although there has been a systematic review on the use of herbal medicine in animal experiments for HFpEF and its potential mechanisms, to the best of our knowledge, this study represents the first systematic review focusing on the effects of herbal medicine on animal experiments exploring post-MI HFrEF. Studies in which the LVEF values of the animals used in the experiments were confirmed to be below 50% were included, reducing heterogeneity among the included studies. Additionally, by reviewing recent theories on HF treatment and examining research gaps, we conducted a review regarding histopathological changes over time following MI induction. This approach is significant because it proposes a new research methodology that considers the pathophysiological timing of drug administration. 

This study has several limitations. All included studies were conducted in China. Some included studies did not describe the exact origin of the composite herbs in the test drugs. Furthermore, it was challenging to identify the active compounds of the test drugs in the included studies. This review was limited to studies using rats or mice as research animals, suggesting the need for future reviews involving larger animals, such as pigs. In addition, the overall risk of bias in the included studies was relatively high.

## 5. Conclusions

A systematic review was conducted on experimental studies in which traditional herbal medicines were administered to post-MI HFrEF murine models with an LVEF of ≤50%. The review aimed to investigate the types of herbal medicines used for the treatment of HFrEF, the formulation of the test drug, study design including the model induction period and treatment duration, and mechanisms and outcomes. This review found that herbs such as *Panax ginseng*, *Astragalus membranaceus*, *Salvia miltiorrhiza*, *Carthamus tinctorius*, and *Lepidium apetalum* were frequently used in test drugs, demonstrating their effects through various signaling pathways, including antifibrosis, anti-inflammation, and anti-apoptosis. Additionally, it highlights the need for future research design considering the time-dependent immunohistological features of the myocardium and lymphatic function according to the disease phase in HF treatment with herbal medicines. 

## Data Availability

The raw data supporting the conclusions of this article will be made available by the authors on request.

## Supplementary Materials

The following supporting information can be downloaded at: https://www.mdpi.com/article/10.3390/medicina60071101/s1, Table S1. Full search strategies and number of identified articles. Table S2. Heart failure animal model induction period after left anterior descending coronary artery ligation and treatment duration in the research design. Table S3. Compositions of herbal medicines of the included studies. Table S4. Comprehensive list of all herbs involved in the herbal medicines of the included studies. Table S5. Compositions of herbal medicines sorted according to the result of the hierarchical cluster analysis. Table S6. Details of design, outcomes, and suggested mechanisms of the included studies.

## Abbreviations

ACC: American College of Cardiology; AHA, American Heart Association; BYTH, Bao Yuan Tao Hong; CL, *Carthamus tinctorius* L. and *Lepidium apetalum* Willd. herbal solution; DQ, Danqi soft capsule; EATM, East Asian traditional medicine; FM, Fumai granule; GBE, *Ginkgo biloba* extract; GDMT, guideline-directed medical therapy; HF, heart failure; HFpEF, heart failure with preserved ejection fraction; HFrEF, heart failure with reduced ejection fraction; HFSA, Heart Failure Society of America; LADCA, left anterior descending coronary artery; LVD, left ventricular dysfunction; LVEF, left ventricular ejection fraction; MI, myocardial infarction; ONSMP, optimized new Shengmai powder; PRISMA, Preferred Reporting Items for Systematic Reviews and Meta-Analyses; QD, Qi Dan Li Xin pill; QL, Qiliqiangxin; QLQX, Qiliqiangxin; QSYQ, Qisheng-Yiqi Dropping Pill; RS-FZ, Renshen-Fuzi herb pair; Sanshen, Sanshen Weixin Capsula; Shenfu, Shenfu Formula; SMY, Shengmai Yin; SSYX, Shengsong Yangxin capsule; SXBXP, Shexiangbaoxin pills; XBP, Xinbao Pill; XG, Xinfuli granule; XLF, XinLi formula; XSB, Xin-shu-bao tablet; YHXSMS, optimized new Shengmai powder; YX, Yixin Granule; RoB, risk of bias; and SYRCLE, Systematic Review Centre for Laboratory Animal Experimentation.
